# Redetermination of chlorido(2,2′:6′,2′′-terpyridine-κ^3^ *N*,*N*′,*N*′′)gold(I) dichloride trihydrate at 173 K

**DOI:** 10.1107/S1600536808027943

**Published:** 2008-09-06

**Authors:** Holger B. Friedrich, Glenn E. M. Maguire, Bice S. Martincigh, Michael G. McKay, Lauren K. Pietersen

**Affiliations:** a School of Chemistry, University of KwaZulu-Natal, Westville Campus, Private Bag X54001, Durban 4000, South Africa

## Abstract

The redetermined structure of the title compound, [AuCl(C_15_H_11_N_3_)]Cl_2_·3H_2_O, at 173 (2) K is reported. The structure displays O—H⋯Cl and O—H⋯O hydrogen bonding. The distance of one of the chloride ions from the gold(I) atom [5.047 (1) Å] differs from that determined previously.

## Related literature

For the previous determination of the crystal structure of the title compound, see: Hollis & Lippard (1983 ▶).
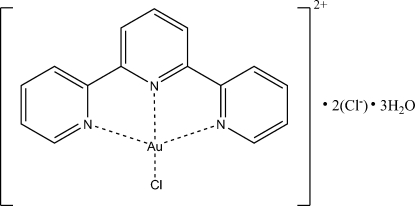


## Experimental

### 

#### Crystal data

[AuCl(C_15_H_11_N_3_)]Cl_2_·3H_2_O
*M* *_r_* = 590.63
Monoclinic, 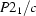

*a* = 8.4486 (1) Å
*b* = 6.9766 (1) Å
*c* = 31.1581 (6) Å
β = 94.392 (1)°
*V* = 1831.14 (5) Å^3^
*Z* = 4
Mo *K*α radiation
μ = 8.49 mm^−1^
*T* = 173 (2) K
0.41 × 0.31 × 0.30 mm

#### Data collection

Bruker APEXII CCD area-detector diffractometer
Absorption correction: integration [Face-indexed absorption corrections carried out with *XPREP* (Bruker, 2005 ▶)] *T*_min_ = 0.128, *T*_max_ = 0.185
13022 measured reflections
4386 independent reflections
4157 reflections with *I* > 2σ(*I*)
*R*_int_ = 0.034

#### Refinement

*R*[*F* ^2^ > 2σ(*F* ^2^)] = 0.033
*wR*(*F* ^2^) = 0.061
*S* = 1.37
4386 reflections
226 parameters
H-atom parameters constrained
Δρ_max_ = 1.32 e Å^−3^
Δρ_min_ = −2.23 e Å^−3^

### 

Data collection: *APEX2* (Bruker, 2005 ▶); cell refinement: *SAINT-NT* (Bruker, 2005 ▶); data reduction: *SAINT-NT*; program(s) used to solve structure: *SHELXTL* (Sheldrick, 2008 ▶); program(s) used to refine structure: *SHELXTL*; molecular graphics: *ORTEP-3* (Farrugia, 1997 ▶); software used to prepare material for publication: *SHELXTL*.

## Supplementary Material

Crystal structure: contains datablocks I, global. DOI: 10.1107/S1600536808027943/ez2137sup1.cif

Structure factors: contains datablocks I. DOI: 10.1107/S1600536808027943/ez2137Isup2.hkl

Additional supplementary materials:  crystallographic information; 3D view; checkCIF report

## Figures and Tables

**Table 1:** Hydrogen-bond geometry (Å, °)

*D*—H⋯*A*	*D*—H	H⋯*A*	*D*⋯*A*	*D*—H⋯*A*
O1*W*—H1*A*⋯Cl2^i^	0.84	2.32	3.140 (4)	167
O1*W*—H1*B*⋯Cl3	0.84	2.42	3.229 (4)	161
O2*W*—H2*A*⋯Cl2	0.84	2.31	3.141 (4)	172
O2*W*—H2*B*⋯O3*W*	0.84	1.94	2.768 (5)	169
O3*W*—H3*A*⋯Cl3^ii^	0.84	2.31	3.133 (4)	167
O3*W*—H3*B*⋯Cl3^iii^	0.84	2.36	3.194 (4)	171

Symmetry codes: (i) 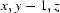; (ii) 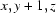; (iii) 
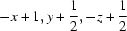
.

**Fig. 1.**
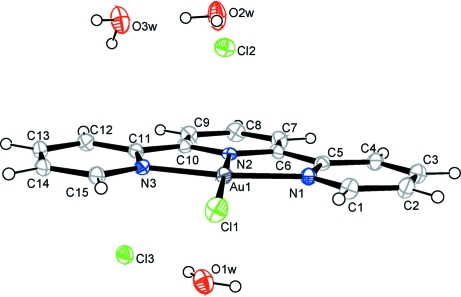
The asymmetric unit of I. Displacement ellipsoids are drawn at the 40% probability level. The labelling of atoms is as shown, and is identical to that of Hollis & Lippard. The two chloride counter ions and the three water molecules associated with I are evident, present above and below the plane of the molecule.

**Fig. 2.**
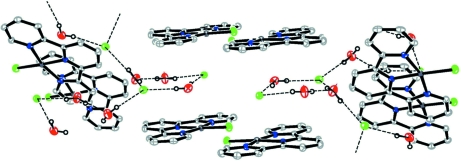
Intermolecular hydrogen bonding present in I, shown by the dashed lines. Hanging dashed lines indicate bonding to an adjacent unit cell. Displacement ellipsoids are drawn at the 40% probability level. Protons belonging to individual molecules have been omitted for purposes of clarity. The water and the chloride ions are seen to be found between the individual molecules of I.

## supplementary crystallographic information

### Comment

The title compound (I, Scheme 1) was synthesized as part of an ongoing study of the DNA binding and intercalation properties of metalloterpyridine complexes.

The asymmetric unit (Fig. 1) consists of the planar terpyridine-AuCl complex with one water molecule and one chloride ion above the plane of the complex, and the remaining two water molecules and a chloride ion below the plane.

A search of the literature revealed that the crystal structure of this compound was previously reported by Hollis & Lippard (1983). The reported structure possesses almost identical crystal parameters to the structure reported here in terms of space group, and unit-cell dimensions and angles. In addition, the coordination sphere parameters appear to closely match those obtained previously. However, the collection of data at 173 K, in comparison to 296 K as reported by Hollis & Lippard, results in some notable conclusions in addition to yielding more accurate data.

The water molecules and chloride ions are involved in extensive intermolecular hydrogen bonding. Fig. 2 illustrates these interactions which extend throughout the crystal structure. The atoms and interaction distances involved in the hydrogen bonding are very similar to those proposed by Hollis & Lippard, and the redetermined hydrogen bonding network confirms the proposed hydrogen bonds.

Additionally, Cl3 is orientated differently here than in the previously determined structure. With reference to the gold centre, Cl3 is found 5.047 (1) Å from the metal atom while the structure reported by Hollis & Lippard shows this distance to be 5.016 Å.

### Experimental

A mixture of AuCl_4_.3H_2_O (0.100 g, 0.29 mmol) and terpyridine (0.072 g, 0.31 mmol) was placed in 10 ml of deionized water in a round-bottom flask. The solution pH was adjusted to 3.0 with 1 *M* NaOH, and the resulting mixture refluxed at 100°C for 24 h. The red mixture was then cooled to room temperature and filtered to remove small amounts of a purple solid. The filtrate obtained was allowed to stand at room temperature to induce precipitation of the crystalline product. The crystals were filtered off and air-dried to yield the title compound. (0.013 g, 7.5%), mp 503 K. ^1^H NMR [DMSO, 600 MHz]: δ = 8.07 (t, 2H), 8.13 (t, 1H), 8.47 (d, 2H), 8.66 (t, 2H), 8.67 (d, 2H), 8.71 (d, 2H). IR (KBr, cm^-1^): 3341, 1604, 1584, 1035. ^13^C NMR [DMSO, 150 MHz]: δ = 151.8, 151.5, 146.8, 141.5, 139.7, 126.0, 122.8, 122.7.

### Refinement

Hydrogen atoms were positioned geometrically and allowed to ride on their respective parent atoms, with C—H bond lengths of 0.93 Å (CH) in a riding model with *U*_iso_(H) = 1.2*U*_eq_(*X*) for *X* = CH.

### Crystal data

**Table:**

[AuCl(C_15_H_11_N_3_)]Cl_2_·3H_2_O	*F*(000) = 1128
*M**_r_* = 590.63	*D*_x_ = 2.142 Mg m^−^^3^
Monoclinic, *P*2_1_/*c*	Mo *K*α radiation, λ = 0.71073 Å
Hall symbol: -P 2ybc	Cell parameters from 8408 reflections
*a* = 8.4486 (1) Å	θ = 3.8–28.3°
*b* = 6.9766 (1) Å	µ = 8.49 mm^−^^1^
*c* = 31.1581 (6) Å	*T* = 173 K
β = 94.392 (1)°	Block, brown
*V* = 1831.14 (5) Å^3^	0.41 × 0.31 × 0.30 mm
*Z* = 4	

### Data collection

**Table:**

Bruker APEXII CCD area-detector diffractometer	4386 independent reflections
Radiation source: fine-focus sealed tube	4157 reflections with *I* > 2σ(*I*)
graphite	*R*_int_ = 0.034
φ and ω scans	θ_max_ = 28.0°, θ_min_ = 3.5°
Absorption correction: integration [Face-indexed absorption corrections carried out with XPREP (Bruker, 2005)]	*h* = −11→11
*T*_min_ = 0.128, *T*_max_ = 0.185	*k* = −9→8
13022 measured reflections	*l* = −41→39

### Refinement

**Table:**

Refinement on *F*^2^	Primary atom site location: structure-invariant direct methods
Least-squares matrix: full	Secondary atom site location: difference Fourier map
*R*[*F*^2^ > 2σ(*F*^2^)] = 0.033	Hydrogen site location: inferred from neighbouring sites
*wR*(*F*^2^) = 0.061	H-atom parameters constrained
*S* = 1.37	*w* = 1/[σ^2^(*F*_o_^2^) + (0.0093*P*)^2^ + 4.719*P*] where *P* = (*F*_o_^2^ + 2*F*_c_^2^)/3
4386 reflections	(Δ/σ)_max_ = 0.001
226 parameters	Δρ_max_ = 1.32 e Å^−^^3^
0 restraints	Δρ_min_ = −2.24 e Å^−^^3^

### Special details

**Table:**

Geometry. All e.s.d.'s (except the e.s.d. in the dihedral angle between two l.s. planes) are estimated using the full covariance matrix. The cell e.s.d.'s are taken into account individually in the estimation of e.s.d.'s in distances, angles and torsion angles; correlations between e.s.d.'s in cell parameters are only used when they are defined by crystal symmetry. An approximate (isotropic) treatment of cell e.s.d.'s is used for estimating e.s.d.'s involving l.s. planes.
Refinement. Refinement of *F*^2^ against ALL reflections. The weighted *R*-factor *wR* and goodness of fit *S* are based on *F*^2^, conventional *R*-factors *R* are based on *F*, with *F* set to zero for negative *F*^2^. The threshold expression of *F*^2^ > σ(*F*^2^) is used only for calculating *R*-factors(gt) *etc*. and is not relevant to the choice of reflections for refinement. *R*-factors based on *F*^2^ are statistically about twice as large as those based on *F*, and *R*- factors based on ALL data will be even larger.

### Fractional atomic coordinates and isotropic or equivalent isotropic displacement parameters (Å^2^)

**Table:**

	*x*	*y*	*z*	*U*_iso_*/*U*_eq_	
C1	−0.0770 (6)	0.2047 (7)	0.02507 (15)	0.0253 (10)	
H1	−0.0237	0.2331	0.0001	0.030*	
C2	−0.2269 (6)	0.1252 (7)	0.02079 (17)	0.0298 (11)	
H2	−0.2766	0.0986	−0.0069	0.036*	
C3	−0.3033 (6)	0.0848 (7)	0.05707 (17)	0.0298 (11)	
H3	−0.4064	0.0297	0.0545	0.036*	
C4	−0.2298 (5)	0.1246 (7)	0.09735 (16)	0.0248 (10)	
H4	−0.2823	0.0976	0.1225	0.030*	
C5	−0.0803 (5)	0.2036 (6)	0.10059 (15)	0.0204 (9)	
C6	0.0097 (5)	0.2552 (6)	0.14095 (14)	0.0196 (9)	
C7	−0.0305 (5)	0.2284 (7)	0.18273 (15)	0.0261 (10)	
H7	−0.1293	0.1721	0.1882	0.031*	
C8	0.0750 (6)	0.2845 (8)	0.21643 (16)	0.0290 (11)	
H8	0.0478	0.2669	0.2452	0.035*	
C9	0.2201 (6)	0.3663 (7)	0.20891 (15)	0.0265 (10)	
H9	0.2926	0.4037	0.2322	0.032*	
C10	0.2570 (5)	0.3922 (6)	0.16673 (15)	0.0203 (9)	
C11	0.4020 (5)	0.4747 (6)	0.15124 (15)	0.0209 (9)	
C12	0.5251 (5)	0.5513 (7)	0.17706 (17)	0.0272 (10)	
H12	0.5194	0.5570	0.2074	0.033*	
C13	0.6570 (5)	0.6197 (7)	0.15853 (18)	0.0305 (11)	
H13	0.7427	0.6729	0.1762	0.037*	
C14	0.6651 (5)	0.6113 (7)	0.11450 (18)	0.0287 (11)	
H14	0.7564	0.6564	0.1016	0.034*	
C15	0.5375 (5)	0.5358 (7)	0.08946 (17)	0.0250 (10)	
H15	0.5408	0.5313	0.0591	0.030*	
Cl1	0.28844 (15)	0.3714 (2)	0.00827 (4)	0.0321 (3)	
Cl2	0.06873 (13)	0.75596 (17)	0.08334 (4)	0.0268 (2)	
Cl3	0.56001 (14)	0.06210 (19)	0.19728 (4)	0.0298 (3)	
Au1	0.211462 (19)	0.35966 (2)	0.076389 (5)	0.01864 (6)	
N1	−0.0062 (4)	0.2421 (5)	0.06372 (12)	0.0206 (8)	
N2	0.1517 (4)	0.3343 (5)	0.13536 (11)	0.0162 (7)	
N3	0.4098 (4)	0.4693 (5)	0.10746 (12)	0.0200 (8)	
O1W	0.3810 (4)	−0.0056 (6)	0.10320 (13)	0.0384 (9)	
H1A	0.3000	−0.0750	0.1021	0.058*	
H1B	0.4060	0.0050	0.1296	0.058*	
O2W	0.0068 (4)	0.7688 (7)	0.18143 (12)	0.0422 (10)	
H2A	0.0261	0.7780	0.1555	0.063*	
H2B	0.0931	0.7980	0.1949	0.063*	
O3W	0.2720 (4)	0.8576 (6)	0.23548 (13)	0.0449 (10)	
H3A	0.3421	0.9059	0.2213	0.067*	
H3B	0.3221	0.7749	0.2508	0.067*	

### Atomic displacement parameters (Å^2^)

**Table:**

	*U*^11^	*U*^22^	*U*^33^	*U*^12^	*U*^13^	*U*^23^
C1	0.027 (2)	0.028 (3)	0.021 (2)	−0.0037 (19)	0.0037 (18)	0.002 (2)
C2	0.028 (2)	0.028 (3)	0.033 (3)	−0.006 (2)	−0.003 (2)	−0.004 (2)
C3	0.024 (2)	0.026 (3)	0.039 (3)	−0.0032 (19)	0.002 (2)	−0.002 (2)
C4	0.021 (2)	0.021 (2)	0.033 (3)	−0.0039 (18)	0.0076 (18)	−0.001 (2)
C5	0.021 (2)	0.015 (2)	0.025 (2)	0.0008 (16)	0.0039 (17)	0.0019 (18)
C6	0.0170 (19)	0.017 (2)	0.026 (2)	0.0012 (16)	0.0065 (17)	0.0039 (18)
C7	0.024 (2)	0.030 (3)	0.025 (2)	−0.0022 (19)	0.0101 (19)	0.001 (2)
C8	0.033 (3)	0.035 (3)	0.019 (2)	0.001 (2)	0.0074 (19)	0.002 (2)
C9	0.027 (2)	0.031 (3)	0.021 (2)	0.002 (2)	0.0013 (18)	−0.001 (2)
C10	0.021 (2)	0.016 (2)	0.024 (2)	0.0010 (16)	0.0032 (17)	0.0017 (18)
C11	0.020 (2)	0.018 (2)	0.025 (2)	0.0020 (16)	0.0048 (17)	0.0004 (18)
C12	0.023 (2)	0.026 (3)	0.032 (3)	0.0007 (19)	−0.0009 (19)	0.000 (2)
C13	0.018 (2)	0.024 (3)	0.049 (3)	−0.0013 (18)	−0.001 (2)	−0.001 (2)
C14	0.018 (2)	0.020 (3)	0.048 (3)	−0.0018 (17)	0.008 (2)	0.001 (2)
C15	0.022 (2)	0.021 (2)	0.033 (3)	−0.0013 (18)	0.0076 (19)	0.001 (2)
Cl1	0.0363 (6)	0.0386 (7)	0.0230 (6)	−0.0093 (5)	0.0120 (5)	−0.0003 (5)
Cl2	0.0285 (5)	0.0258 (6)	0.0260 (6)	0.0037 (4)	0.0015 (4)	0.0004 (5)
Cl3	0.0263 (5)	0.0290 (6)	0.0344 (6)	−0.0015 (5)	0.0044 (5)	0.0023 (5)
Au1	0.01834 (8)	0.01925 (9)	0.01896 (9)	−0.00206 (7)	0.00558 (6)	0.00098 (7)
N1	0.0188 (17)	0.019 (2)	0.024 (2)	−0.0018 (14)	0.0045 (15)	0.0004 (16)
N2	0.0180 (16)	0.0136 (18)	0.0175 (17)	0.0029 (13)	0.0040 (13)	0.0000 (14)
N3	0.0181 (17)	0.0163 (19)	0.026 (2)	−0.0003 (14)	0.0027 (15)	0.0003 (16)
O1W	0.0303 (19)	0.043 (2)	0.042 (2)	−0.0015 (17)	0.0029 (16)	0.0090 (19)
O2W	0.0289 (18)	0.068 (3)	0.030 (2)	−0.0122 (19)	0.0052 (15)	−0.003 (2)
O3W	0.0305 (19)	0.061 (3)	0.042 (2)	−0.0077 (19)	−0.0007 (17)	0.013 (2)

### Geometric parameters (Å, °)

**Table:**

C1—N1	1.329 (6)	C11—N3	1.371 (6)
C1—C2	1.380 (6)	C11—C12	1.373 (7)
C1—H1	0.9500	C12—C13	1.379 (7)
C2—C3	1.373 (7)	C12—H12	0.9500
C2—H2	0.9500	C13—C14	1.380 (8)
C3—C4	1.385 (7)	C13—H13	0.9500
C3—H3	0.9500	C14—C15	1.386 (7)
C4—C5	1.375 (6)	C14—H14	0.9500
C4—H4	0.9500	C15—N3	1.336 (5)
C5—N1	1.376 (6)	C15—H15	0.9500
C5—C6	1.463 (6)	Cl1—Au1	2.2686 (11)
C6—N2	1.345 (5)	Au1—N2	1.950 (3)
C6—C7	1.383 (6)	Au1—N3	2.021 (4)
C7—C8	1.381 (7)	Au1—N1	2.025 (4)
C7—H7	0.9500	O1W—H1A	0.8369
C8—C9	1.388 (7)	O1W—H1B	0.8373
C8—H8	0.9500	O2W—H2A	0.8388
C9—C10	1.386 (6)	O2W—H2B	0.8381
C9—H9	0.9500	O3W—H3A	0.8361
C10—N2	1.333 (6)	O3W—H3B	0.8423
C10—C11	1.468 (6)		
			
N1—C1—C2	120.8 (4)	C12—C11—C10	125.0 (4)
N1—C1—H1	119.6	C11—C12—C13	119.3 (5)
C2—C1—H1	119.6	C11—C12—H12	120.3
C3—C2—C1	119.3 (5)	C13—C12—H12	120.3
C3—C2—H2	120.4	C12—C13—C14	120.3 (5)
C1—C2—H2	120.4	C12—C13—H13	119.8
C2—C3—C4	119.9 (5)	C14—C13—H13	119.8
C2—C3—H3	120.1	C13—C14—C15	118.7 (4)
C4—C3—H3	120.1	C13—C14—H14	120.7
C5—C4—C3	119.5 (4)	C15—C14—H14	120.7
C5—C4—H4	120.2	N3—C15—C14	120.9 (5)
C3—C4—H4	120.2	N3—C15—H15	119.5
C4—C5—N1	119.4 (4)	C14—C15—H15	119.5
C4—C5—C6	125.1 (4)	N2—Au1—N3	81.25 (15)
N1—C5—C6	115.5 (4)	N2—Au1—N1	81.39 (15)
N2—C6—C7	117.6 (4)	N3—Au1—N1	162.64 (15)
N2—C6—C5	113.6 (4)	N2—Au1—Cl1	176.48 (11)
C7—C6—C5	128.9 (4)	N3—Au1—Cl1	98.51 (11)
C8—C7—C6	119.2 (4)	N1—Au1—Cl1	98.82 (11)
C8—C7—H7	120.4	C1—N1—C5	121.1 (4)
C6—C7—H7	120.4	C1—N1—Au1	126.5 (3)
C7—C8—C9	121.0 (4)	C5—N1—Au1	112.4 (3)
C7—C8—H8	119.5	C10—N2—C6	125.6 (4)
C9—C8—H8	119.5	C10—N2—Au1	117.2 (3)
C10—C9—C8	118.7 (4)	C6—N2—Au1	117.2 (3)
C10—C9—H9	120.7	C15—N3—C11	120.7 (4)
C8—C9—H9	120.7	C15—N3—Au1	126.7 (3)
N2—C10—C9	118.0 (4)	C11—N3—Au1	112.7 (3)
N2—C10—C11	113.9 (4)	H1A—O1W—H1B	103.6
C9—C10—C11	128.1 (4)	H2A—O2W—H2B	103.8
N3—C11—C12	120.1 (4)	H3A—O3W—H3B	103.3
N3—C11—C10	114.9 (4)		
			
N1—C1—C2—C3	0.1 (8)	N2—Au1—N1—C1	178.4 (4)
C1—C2—C3—C4	0.2 (8)	N3—Au1—N1—C1	178.2 (4)
C2—C3—C4—C5	−0.3 (7)	Cl1—Au1—N1—C1	−5.1 (4)
C3—C4—C5—N1	0.1 (7)	N2—Au1—N1—C5	−0.9 (3)
C3—C4—C5—C6	179.1 (4)	N3—Au1—N1—C5	−1.1 (7)
C4—C5—C6—N2	−178.5 (4)	Cl1—Au1—N1—C5	175.6 (3)
N1—C5—C6—N2	0.5 (6)	C9—C10—N2—C6	1.3 (7)
C4—C5—C6—C7	3.4 (8)	C11—C10—N2—C6	−179.6 (4)
N1—C5—C6—C7	−177.6 (5)	C9—C10—N2—Au1	−176.8 (3)
N2—C6—C7—C8	0.5 (7)	C11—C10—N2—Au1	2.3 (5)
C5—C6—C7—C8	178.5 (5)	C7—C6—N2—C10	−1.1 (7)
C6—C7—C8—C9	−0.2 (8)	C5—C6—N2—C10	−179.4 (4)
C7—C8—C9—C10	0.5 (8)	C7—C6—N2—Au1	177.0 (3)
C8—C9—C10—N2	−1.0 (7)	C5—C6—N2—Au1	−1.3 (5)
C8—C9—C10—C11	−179.9 (5)	N3—Au1—N2—C10	−0.6 (3)
N2—C10—C11—N3	−3.5 (6)	N1—Au1—N2—C10	179.5 (3)
C9—C10—C11—N3	175.5 (4)	N3—Au1—N2—C6	−178.9 (3)
N2—C10—C11—C12	177.3 (4)	N1—Au1—N2—C6	1.2 (3)
C9—C10—C11—C12	−3.7 (8)	C14—C15—N3—C11	0.2 (7)
N3—C11—C12—C13	−0.9 (7)	C14—C15—N3—Au1	178.7 (3)
C10—C11—C12—C13	178.2 (4)	C12—C11—N3—C15	0.9 (7)
C11—C12—C13—C14	0.0 (7)	C10—C11—N3—C15	−178.4 (4)
C12—C13—C14—C15	1.0 (7)	C12—C11—N3—Au1	−177.8 (4)
C13—C14—C15—N3	−1.1 (7)	C10—C11—N3—Au1	2.9 (5)
C2—C1—N1—C5	−0.3 (7)	N2—Au1—N3—C15	−179.9 (4)
C2—C1—N1—Au1	−179.6 (4)	N1—Au1—N3—C15	−179.7 (4)
C4—C5—N1—C1	0.2 (7)	Cl1—Au1—N3—C15	3.6 (4)
C6—C5—N1—C1	−178.9 (4)	N2—Au1—N3—C11	−1.4 (3)
C4—C5—N1—Au1	179.5 (3)	N1—Au1—N3—C11	−1.1 (7)
C6—C5—N1—Au1	0.5 (5)	Cl1—Au1—N3—C11	−177.8 (3)

### Hydrogen-bond geometry (Å, °)

**Table:**

*D*—H···*A*	*D*—H	H···*A*	*D*···*A*	*D*—H···*A*
O1W—H1A···Cl2^i^	0.84	2.32	3.140 (4)	167
O1W—H1B···Cl3	0.84	2.42	3.229 (4)	161
O2W—H2A···Cl2	0.84	2.31	3.141 (4)	172
O2W—H2B···O3W	0.84	1.94	2.768 (5)	169
O3W—H3A···Cl3^ii^	0.84	2.31	3.133 (4)	167
O3W—H3B···Cl3^iii^	0.84	2.36	3.194 (4)	171

Symmetry codes: (i) *x*, *y*−1, *z*; (ii) *x*, *y*+1, *z*; (iii) −*x*+1, *y*+1/2, −*z*+1/2.
