# Redetermination of chlorido(2,2′:6′,2′′-terpyridine-κ^3^ *N*,*N*′,*N*′′)gold(I) dichloride trihydrate at 173 K. Corrigendum

**DOI:** 10.1107/S1600536809016122

**Published:** 2009-05-07

**Authors:** Holger B. Friedrich, Glenn E. M. Maguire, Bice S. Martincigh, Michael G. McKay, Lauren K. Pietersen

**Affiliations:** a School of Chemistry, University of KwaZulu-Natal, Westville Campus, Private Bag X54001, Durban 4000, South Africa

## Abstract

The title of the paper by Friedrich, Maguire, Martincigh, McKay & Pietersen [*Acta Cryst.* (2008), E**64**, m1240] is corrected.

## Abstract 2

Corrigendum to *Acta Cryst.* (2008), E**64**, m1240.

In the title of the paper by Friedrich, Maguire, Martincigh, McKay & Pietersen [*Acta Cryst.* (2008), E**64**, m1240], the oxidation state of the Au atom is given incorrectly. The correct title should be ‘Redetermination of chlorido(2,2′:6′,2′′-terpyridine-κ^3^ *N*,*N*′,*N*′′)gold(III) dichloride trihydrate at 173 K’.

